# Effects of interstimulus interval and significance on electrodermal and central measures of the phasic orienting reflex (OR) in a dishabituation task

**DOI:** 10.1038/s41598-023-40428-7

**Published:** 2023-08-19

**Authors:** Robert J. Barry, Genevieve Z. Steiner-Lim, Adele E. Cave, Frances M. De Blasio, Brett MacDonald

**Affiliations:** 1 Brain and Behaviour Research Institute and School of Psychology, University of Wollongong, Wollongong, Australia; 2 NICM Health Research Institute, Western Sydney University, Penrith, Australia

**Keywords:** Neuroscience, Psychology

## Abstract

Although the P300 event-related potential (ERP) is the most likely central measure of Sokolov’s Orienting Reflex (OR), there are few systematic comparisons with the skin conductance response (SCR), the “gold standard” electrodermal OR measure. We examine habituation, stimulus significance, and inter-stimulus interval (ISI) effects in SCRs and components of the P300 from single-trial ERPs in an auditory dishabituation paradigm. Single trial ERP components were separated by temporal principal components analysis, and five components of the P300 were examined as potential phasic OR measures: P3a, P3b, Novelty P3, and two Slow Waves (SW1, SW2). Across the factors of ISI and significance, SCRs showed decrement over trials, recovery at a deviant, and dishabituation at the subsequent standard. This general pattern was not present in any of the components of the P300. SCRs were also larger to significant stimuli and at the long ISI; effects differed between P300 components. The electrodermal SCR showed the complete profile over trials expected of the phasic OR, and was enhanced by stimulus significance, confirming it as the model measure of Sokolov’s phasic OR. Components of the P300 failed to match this profile, but instead appear to reflect different aspects of the stimulus processing involved in OR elicitation.

## Introduction

The Orienting Reflex (OR) is an adaptive mechanism that focuses an organism’s attention towards changes in the environment to facilitate optimal perceptual processing. The founder of the psychophysiology of the OR, E.N. Sokolov, proposed that for a physiological response to be considered as a measure of the OR, it should demonstrate habituation and sensitivity to stimulus characteristics (particularly novelty and significance)^1–3^. Habituation is an important fundamental learning mechanism that enables an organism to maintain homeostasis by reflexively inhibiting responses to irrelevant stimuli. Criteria originally proposed by Thompson and Spencer^4^, and later revised by Rankin and colleagues^5^, specify that for response decrement to be formally labelled as habituation, response recovery (enhanced responding to a change/deviant stimulus) and dishabituation (increased responding to the habituated stimulus following the deviant), must be evident. These criteria are essential for differentiating habituation from other decrementing response patterns, such as those due to refractory periods.

The skin conductance response (SCR), which reflects bursts in sympathetic sudomotor nerve signalling^6^, is considered the “yardstick” or gold standard measure of the OR in humans^7–10^. Due to the slow onset, rise-time, and resolution of the SCR, paradigms with long interstimulus intervals (ISIs) of 10–60 s have been considered necessary to separate SCRs between stimuli^11–14^. However, lengthy ISI studies are incongruous with contemporary psychophysiological paradigms, particularly those designed to elicit event-related potentials (ERPs), relying on averaging responses elicited at short ISIs (~ 1 s duration); here single trials have been thought undesirable due to the poor signal-to-noise ratio^15^. In the quest to find a central nervous system index of the OR, research efforts have been made to elicit SCRs at shorter ISIs in a range of contexts^12,15–18^, and to assess corresponding ERP components as potential OR candidates^18,19^.

Gatchel and Lang^20^ investigated SCRs in an auditory habituation task with mean ISIs of 20, 60, and 100 s and reported smaller SCRs that habituated more rapidly with shorter ISIs. Berti and colleagues^21^ recorded SCRs and ERPs during a two-stimulus auditory oddball task (novel target probability = 10%) at 0.5, 1.0, 3.0, and 10.0 s ISIs in younger and older adults. SCRs and target N2 and P3 ERP component amplitudes decreased with shorter ISIs in both groups. Recio and colleagues^17^ assessed differences in SCR and Go/NoGo ERP components N2 and P3 whilst manipulating ISI to 2, 5, and 8 s. Increasing the ISI enhanced SCRs, but did not alter the condition-specific effects. The same pattern was demonstrated by Breska and colleagues^12^ using a concealed information test to compare SCRs elicited to mean ISIs of 10 and 20 s. They concluded that the ISI should be selected carefully depending on the aims of the study, indeed an important point demonstrated in Steiner and Barry^13,14^. In our 2011 investigation into the mechanism of electrodermal dishabituation^22^, stimulus-onset asynchrony (SOA) at 5–7 s was too short to allow the complete resolution of the phasic SCR to significant (counted) stimuli. Our follow-up investigation^14^ found that an SOA of 13–15 s was adequate for SCR resolution (consistent with Barry^23^).

As Sokolov^1^ noted, significant (task-relevant/salient) stimuli elicit larger ORs that are slower to habituate in the context of elevated arousal levels, an observation confirmed in our previous investigations^13,14,22–24^. We have also suggested that ISI affects stimulus novelty^13,14^, with stimuli presented at shorter ISIs perceived as less novel than at longer ISIs, exhibiting SCRs with smaller amplitudes that decrement more quickly^12–14,17,20^. This may be a potential mechanism for the well-documented P300-ISI effect, where shorter ISIs elicit P300 ERP components with smaller amplitudes and shorter latencies compared to longer ISIs^25–31^.

The P300 (Late Positive Complex, LPC) has long been considered the central analogue of the OR^19,32,33^. This view arose when peak-picking quantification methods were most ubiquitous, where P300 and its underlying components (P3a, P3b, Novelty P3, and at least one Slow Wave [SW]) were collectively measured as a single entity and shown to mirror the SCR, as demonstrated (for example) by Rushby and colleagues^18^. However, once temporal principal components analysis (PCA) is applied to separate temporally overlapping components, not one of the individual members of the “P3 family” are found to parallel the SCR as an OR index^18^. Each P3 component differs in scalp topography, peak latency, and sensitivity to task requirements and stimulus parameters, indicating multi-layered cognitive and perceptual processes constituting the OR, with differential cortical sources to match^8–10,34–38^. We have previously reported response decrement for P3b and Novelty P3 (nP3), response recovery for nP3 only, no components showing dishabituation, and sensitivity to stimulus significance for P3b and SW1 only^9,35,38^. To the best of our awareness, ISI has not yet been explored in this context.

Here, we varied the ISI to explore whether the rate of stimulus presentation affected stimulus novelty and the OR, in the context of indifferent (no task instructions) and significant (counting) stimuli. We used an autonomic-style dishabituation paradigm with two different ISIs (short: 5–7 s; long: 13–15 s) and quantified single trial SCRs and components of the P300, the latter using PCA. It was hypothesised that SCR would “behave” as a typical OR measure: demonstrating habituation, larger responses to significant than indifferent stimuli, and smaller responses that decremented more quickly to short than long ISIs. Corresponding effects were expected to be seen in P300 ERP components in line with our previous investigations^9,35^, with response decrement seen for P3b and nP3, and response recovery and sensitivity to ISI for nP3 only; stimulus significance was expected to enhance P3b and SW1^38^. The later SW2 has not been examined in this context before.

## Results

All participants correctly reported their number of trials to the experimenter at the end of the counting condition, confirming compliance with the task.

### Electrodermal responses

**Figure 1:**
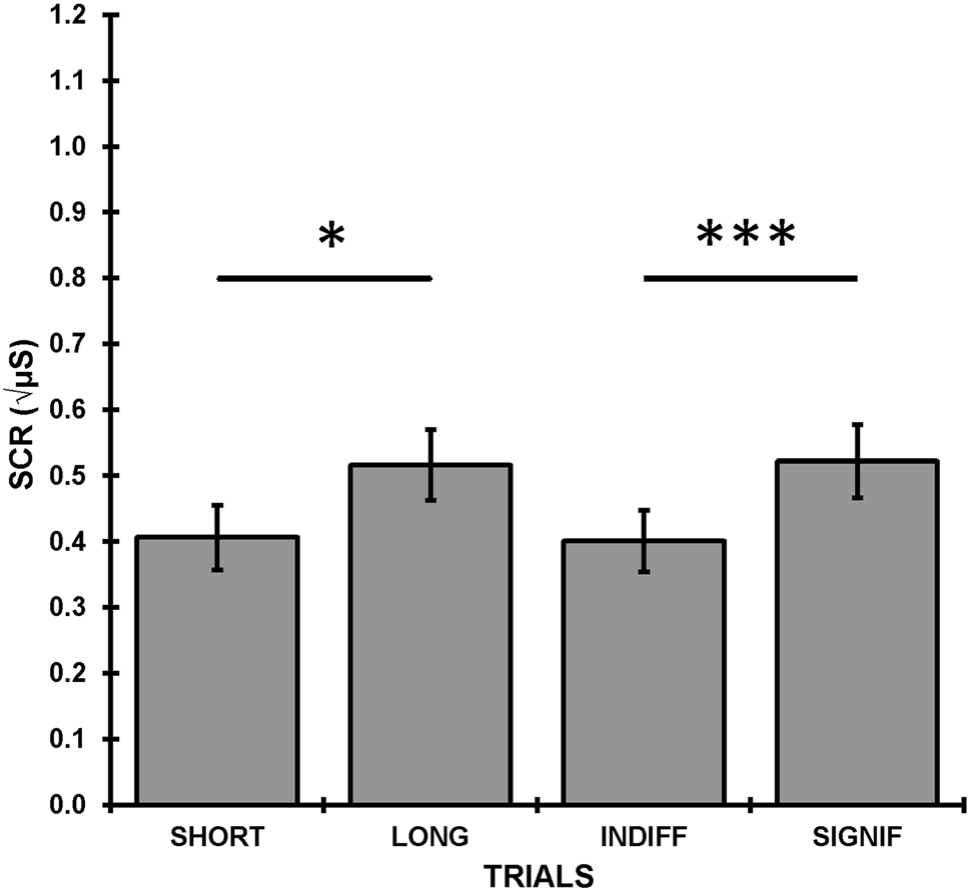
Grand means over trials of skin conductance responses for each inter-stimulus interval and condition. *Indicates significant difference at *p* < 0.05; ***indicates *p* < 0.001.

Figure 1 shows the square-root SCRs averaged over the 12 trials as a function of significance (significant vs. indifferent) and ISI (short vs. long). Responses to significant stimuli were larger than to indifferent stimuli (*F*(1, 38) = 28.29, *p* < 0.001, η_p_^2^ = 0.43), and responses in the long group were larger than in the short group (*F* = 4.35, *p* = 0.044, η_p_^2^ = 0.10). There was no interaction between significance and ISI.

#### Trials effects in electrodermal responses

**Figure 2:**
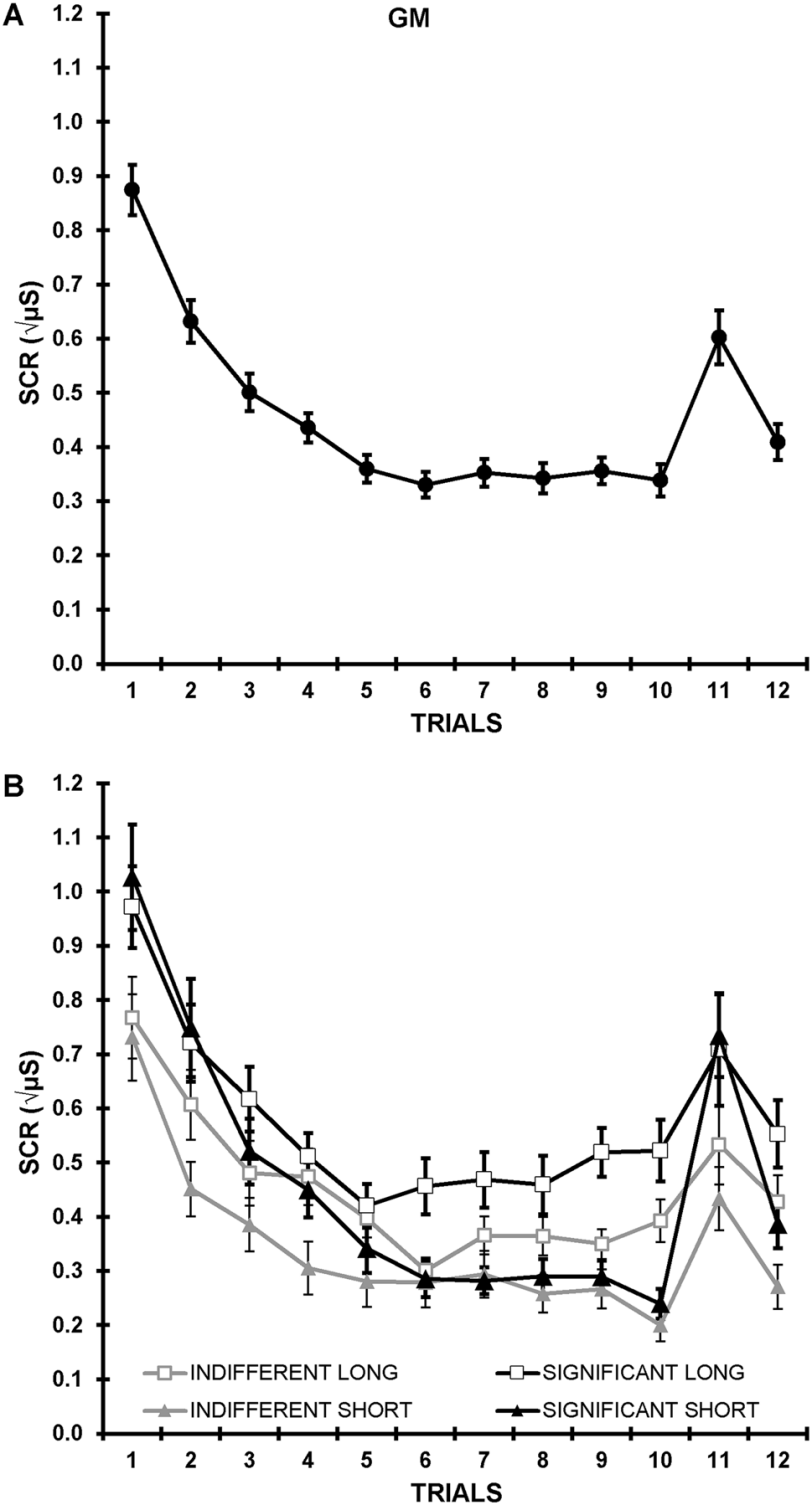
Grand means for skin conductance responses at each trial. (**A**) Across significance and inter-stimulus intervals; (**B**) For each combination of condition and inter-stimulus interval.

Square-root SCRs, averaged across significance and ISI, are shown at each trial in Fig. 2A. Overall, a substantial decrement is apparent over the repeated stimuli. This is interrupted by response recovery to the change stimulus at trial 11. Subsequently, SCRs at trial 12 appear larger than at trial 10 (dishabituation). Figure 2B shows this response profile for each level of significance at each ISI.

Statistically, over trials 1 to 10, decrement was apparent in linear (*F* = 233.00, *p* < 0.001, η_p_^2^ = 0.86), and quadratic (*F* = 127.24, *p* < 0.001, η_p_^2^ = 0.77) trends. Responses were initially larger in the significant versus indifferent condition, leading to greater decrement in both linear (*F* = 5.30, *p* = 0.027, η_p_^2^ = 0.12) and quadratic (*F* = 7.60, *p* = 0.009, η_p_^2^ = 0.17) trends. Greater decrement in the short *cf.* long group was apparent in a linear trend difference (*F* = 9.25, *p* = 0.004, η_p_^2^ = 0.20). There was an interaction between significance and ISI, with the linear decrement being greatest to significant stimuli at the short ISI (*F* = 4.65, *p* = 0.038, η_p_^2^ = 0.11).

Across trials 10 and 11, there was recovery at the change tone (*F* = 48.68, *p* < 0.001, η_p_^2^ = 0.56). Recovery was larger for the significant condition (*F* = 9.14, *p* = 0.004, η_p_^2^ = 0.19), and for the short *cf.* long ISI (*F* = 7.05, *p* = 0.012, η_p_^2^ = 0.16). These interacted, with the significance enhancement larger at the short ISI (*F* = 4.47, *p* = 0.041, η_p_^2^ = 0.11).

Across trials 10 and 12, dishabituation was apparent (*F* = 13.06, *p* < 0.001, η_p_^2^ = 0.26), but this did not differ with significance. Dishabituation was somewhat larger at short ISI (*F* = 3.78, *p* = 0.059, η_p_^2^ = 0.09), but there was no interaction of this with significance.

### ERP components

**Figure 3:**
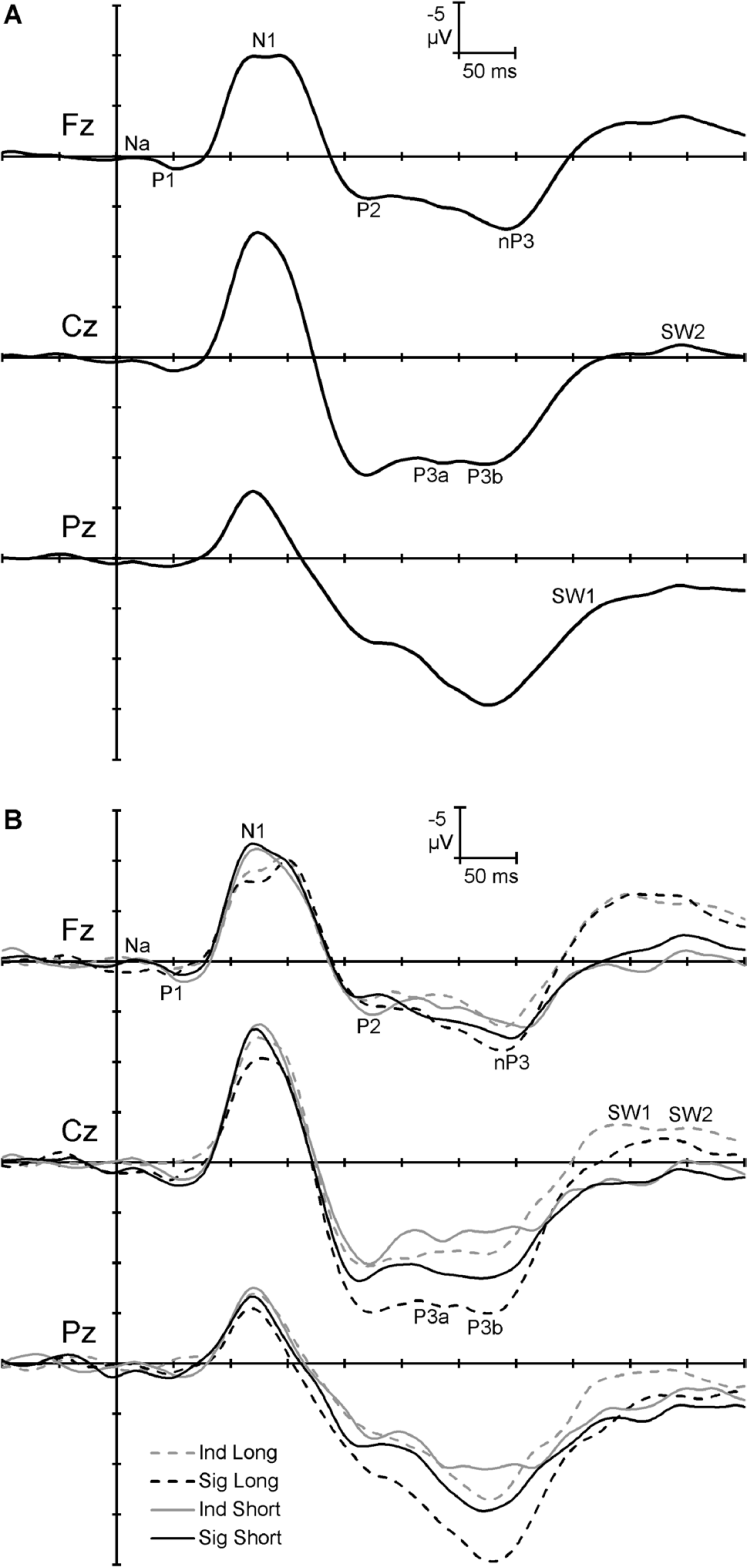
Grand mean ERPs at each of the midline sites. (**A**) Across condition and inter-stimulus intervals; (**B**) For each combination of condition and inter-stimulus interval.

Figure 3A shows the grand mean ERPs at the midline sites averaged over trials, significance, and ISI. There was a small Na and P1, followed by a large N1 complex and P2. A large P3 complex followed, then two slow waves (SW1 and SW2). Figure 3B shows the morphology at the midline sites for each combination of condition and ISI.

**Figure 4:**
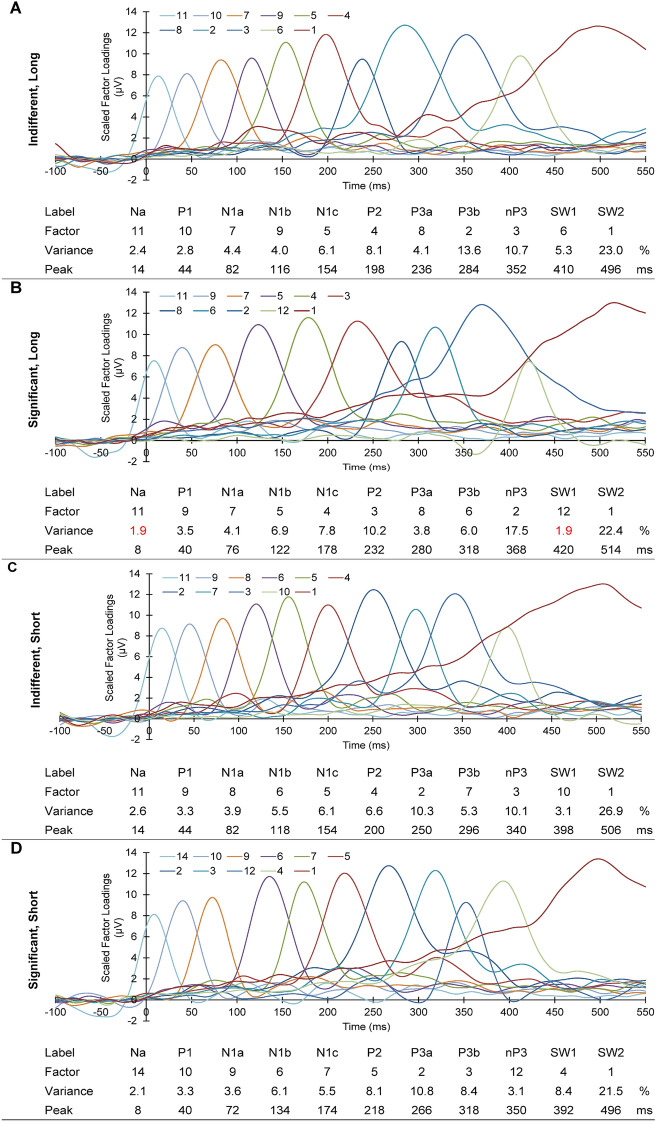
Scaled factor loadings from each of the four PCAs. Corresponding components are shown with lines of the same colour. Below each loadings set are shown the label, factor number, % variance, and peak latency for each component.

**Figure 5:**
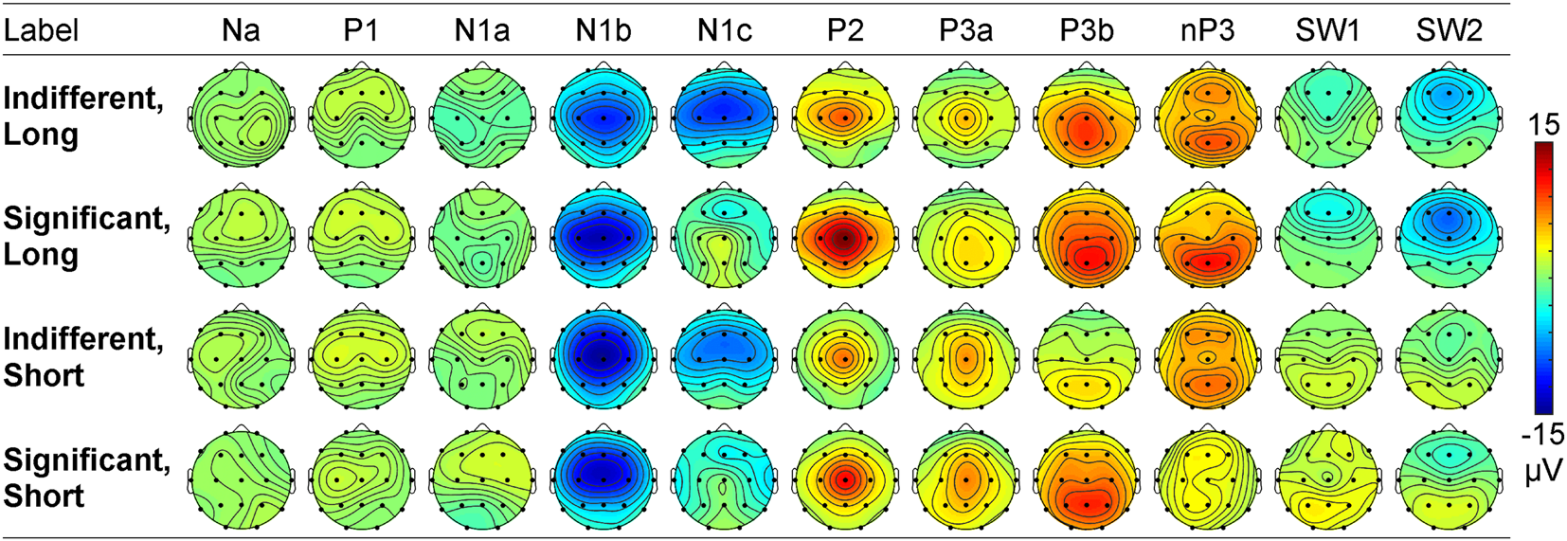
Component headmaps for each combination of condition and inter-stimulus interval.

As shown in Fig. 4, a similar series of components was apparent in each separate PCA, identifiable as: Na, P1, N1a, N1b, N1c, P2, P3a, P3b, nP3, SW1 and SW2. Figure 4A shows that, in the long indifferent PCA, these 11 components carried 84.4% of the variance. In the long significant PCA (Fig. 4B), Na and SW1 emerged only when the threshold was reduced from 2.0% to 1.9%; note variance values in red font. Factor 10 was an unidentifiable prestimulus component at − 18 ms; its omission left 85.8% variance. The short indifferent PCA (Fig. 4C) produced 11 comparable components, carrying 83.7% of the variance. The PCA of the short significant data set (Fig. 4D) produced 11 similar components, carrying 80.7% of the variance. There were also three unidentifiable components, at − 82 ms (i.e., prestimulus), 102, and 436 ms; these were omitted from further consideration. The topographies of the accepted components from the four PCAs are shown in Fig. 5.

**Figure 6:**
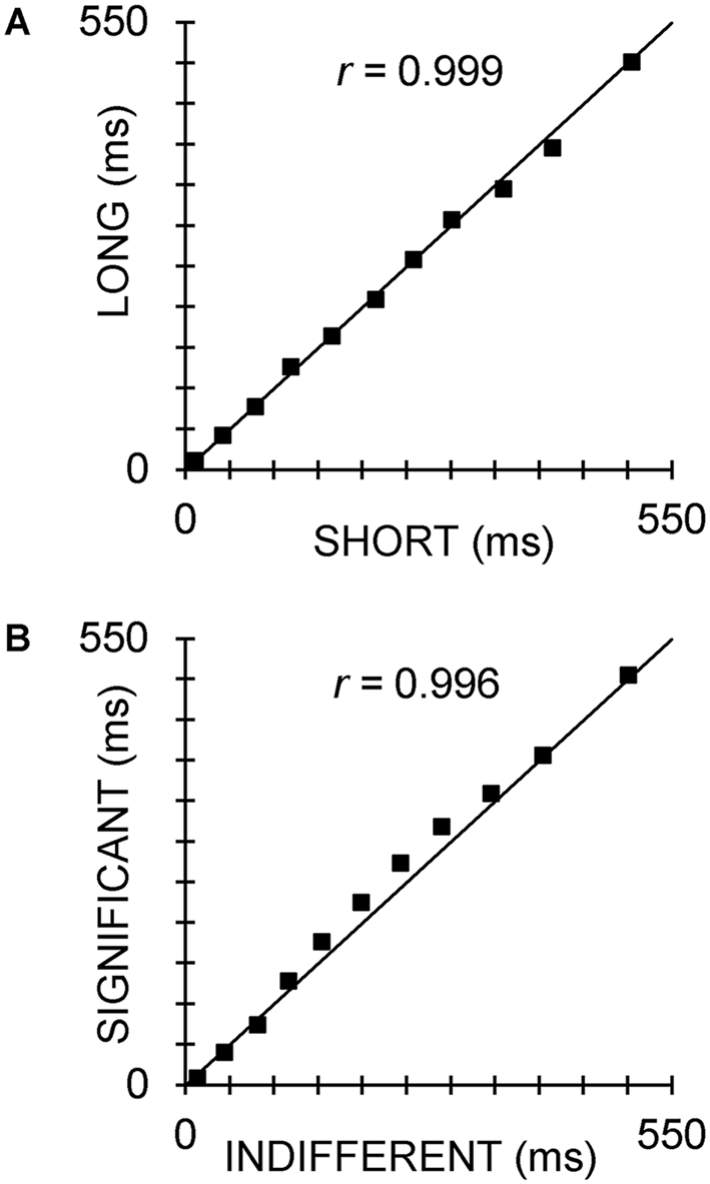
Scatter plots of ERP latencies, with the line of equality included. (**A**) For long versus short ISIs; (**B**) For significant versus indifferent conditions.

The four sets of peak latencies of these corresponding components were closely related. Averaged across significance, Fig. 6A shows that latencies of the long group components correlated with those of the short group components (*r* = 0.999, *p* < 0.0001), and demonstrated a non-significant mean increase of some 3.3 ms (matched samples t-test: *t* = 1.34, two-way *p* = 0.210). Averaged across ISI, Fig. 6B shows that the latencies of the significant condition components correlated with those of the indifferent condition components (*r* = 0.996, *p* < 0.0001), with a significant mean increase of some 10.7 ms (matched samples t-test: *t* = − 1.51, two-way *p* = 0.031). The components contributing to this increase in latency with significance (i.e., those clearly above the line of equality in Fig. 5B) were N1b, N1c, P2, P3a, P3b, and nP3.

Usually, ERP amplitudes are calculated from a region of interest (ROI) reflecting the topography of the component. Here the four sets of topographies (shown in Fig. 5) differed somewhat with significance and ISI. To avoid the complication of different ROIs, we used the mean of the across-scalp amplitudes for each component. The mean of this over all trials was examined as a function of significance and ISI. Na and P1 showed no significant difference between significant and indifferent conditions, no difference between long and short ISI, and no interaction effects. N1a was somewhat larger at the long ISI (*F* = 3.05, *p* = 0.089, η_p_^2^ = 0.07), but showed no other effects. In N1b there were no main effects of significance or ISI, but these interacted significantly, with an increase in the significant *cf.* indifferent condition that was larger for the long ISI group (*F* = 4.48, *p* = 0.041, η_p_^2^ = 0.11). N1c showed a reduction in amplitude with significant *cf.* indifferent stimuli (*F* = 27.37, *p* < 0.001, η_p_^2^ = 0.42), and this was somewhat larger at long ISI (*F* = 3.30, *p* = 0.077, η_p_^2^ = 0.08).

P2 was larger in significant than indifferent conditions (*F* = 6.93, *p* = 0.012, η_p_^2^ = 0.15), and was somewhat larger with long *cf.* short ISIs (*F* = 4.06, *p* = 0.051, η_p_^2^ = 0.10); these effects did not interact. P3a did not vary across significance or ISI. P3b was larger in significant than indifferent conditions (*F* = 13.35, *p* = 0.001, η_p_^2^ = 0.26), and larger at the long than short ISI (*F* = 4.69, *p* = 0.037, η_p_^2^ = 0.11); these effects did not interact. nP3 was reduced somewhat in significant compared to the indifferent condition (*F* = 4.04, *p* = 0.052, η_p_^2^ = 0.10), and was larger at long than short ISI (*F* = 5.71, *p* = 0.022, η_p_^2^ = 0.13); these interacted, with the significant condition reduction larger at the short ISI (*F* = 6.73, *p* = 0.013, η_p_^2^ = 0.15).

The positive SW1 was larger for the short than long ISI group (*F* = 21.55, *p* < 0.001, η_p_^2^ = 0.36), and this effect was larger with significant than indifferent stimuli (*F* = 4.54, *p* = 0.040, η_p_^2^ = 0.11). SW2 was more positive at short *cf.* the long ISI (*F* = 10.99, *p* = 0.002, η_p_^2^ = 0.22).

#### Trials effects in LPC components

**Figure 7:**
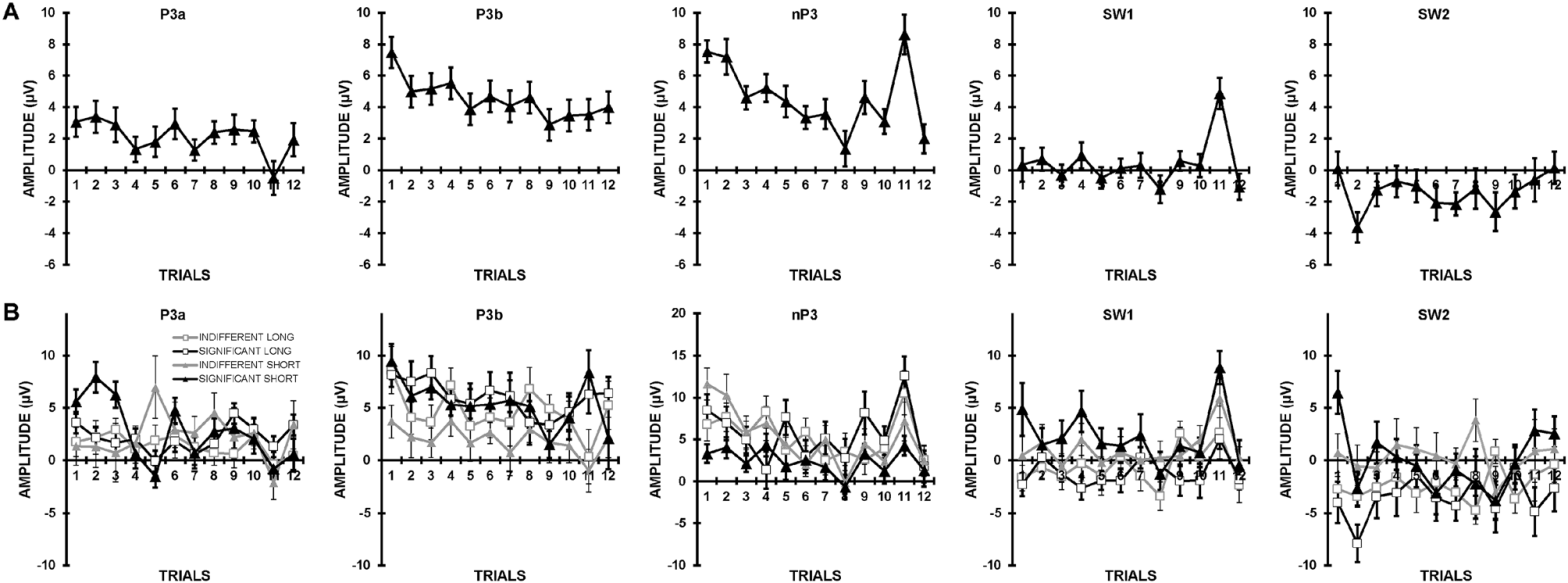
Grand means at each trial for each of the LPC components. (**A**) Across groups and conditions; (**B**) For each condition and inter-stimulus interval combination.

Trials effects in the LPC components (P3a, P3b, nP3, SW1, SW2) are reported here as potential OR correlates. Amplitudes are plotted over trials in Fig. 7a for the mean across groups and conditions, and in Fig. 7b for the individual datasets. P3a showed no overall decrement over trials 1 to 10, but had a faster *initial* decrement for significant stimuli, apparent in a greater quadratic trend over trials (*F* = 5.75, *p* = 0.021, η_p_^2^ = 0.13), and the linear decrement was largest for significant stimuli at the short ISI (*F* = 5.35, *p* = 0.026, η_p_^2^ = 0.12). P3b showed a significant linear decrement (*F* = 10.68, *p* = 0.002, η_p_^2^ = 0.22) that was somewhat larger for significant than indifferent conditions (*F* = 3.39, *p* = 0.073, η_p_^2^ = 0.08). nP3 decremented over trials, apparent in significant linear (*F* = 26.32, *p* < 0.001, η_p_^2^ = 0.41) and quadratic (*F* = 6.18, *p* = 0.017, η_p_^2^ = 0.14) trends; the linear trend was larger for the indifferent than significant condition (*F* = 5.33, *p* = 0.026, η_p_^2^ = 0.12). For the positive SW1, the linear decrement over trials was somewhat greater for significant than indifferent conditions (*F* = 3.57, *p* = 0.066, η_p_^2^ = 0.09). The SW2 positivity showed greater linear decrement over trials at short than long ISI (*F* = 5.18, *p* = 0.029, η_p_^2^ = 0.12), particularly in the significant condition (*F* = 4.16, *p* = 0.048, η_p_^2^ = 0.10).

As shown in Fig. 7A, P3a showed no evidence of recovery at trial 11 *cf.* trial 10; rather, a reduction reached statistical significance (*F* = 5.09, *p* = 0.030, η_p_^2^ = 0.12). Figure 7B shows how P3b had greater recovery in significant than indifferent conditions (*F* = 5.51, *p* = 0.024, η_p_^2^ = 0.13). nP3 showed strong recovery (*F* = 12.99, *p* = 0.001, η_p_^2^ = 0.25) that was independent of significance and ISI. SW1 positivity showed significantly greater response at the change (*F* = 18.99, *p* < 0.001, η_p_^2^ = 0.33) that was independent of stimulus significance and ISI. SW2 positivity showed no recovery at the change. Dishabituation at trial 12 *cf.* trial 10 was not apparent in P3a, P3b, nP3, SW1 or SW2.

### Similarities between SCR and P300 component trials effects

Over the 12 trials, the Spearman correlation between mean P3a trials effects (Fig. 7A) and mean SCR trials effects (Fig. 2A) was not significant, *r*(10) = 0.15, one-tailed *p* = 0.325. However, exploratory analyses indicated that this correlation reached statistical significance for significant stimuli at the long ISI, *r*(10) = 0.50, one-tailed *p* = 0.050, but was negative for the indifferent condition at the short ISI, *r*(10) = − 0.69, two-tailed *p* = 0.014; compare plots in Figs. 7B and 2B. Illustrative scatterplots are presented in Supplementary Fig. S1.

For P3b, the correlation between mean ERP (Fig. 7A) and mean SCR trials effects (Fig. 2A) approached significance, *r*(10) = 0.45, one-tailed *p* = 0.069. Exploratory analyses of the individual condition data in Figs. 7B and 2B indicated that this correlation reached statistical significance in the significant stimulus condition at both short, *r*(10) = 0.64, one-tailed *p* = 0.012, and long ISIs, *r*(10) = 0.57, one-tailed *p* = 0.027. See scatterplots in Supplementary Fig. S2.

With nP3, the correlation between the ERP profile (Fig. 7A) and mean SCR trials effects (Fig. 2A) was significant, *r*(10) = 0.79, one-tailed *p* = 0.001. For the separate condition-specific data in Figs. 7B and 2b, this correlation reached statistical significance in the indifferent condition at both short and long ISIs; both *r*(10) ≥ 0.60, one-tailed *p* ≤ 0.019, and in the significant condition at the short ISI, *r*(10) = 0.63, one-tailed *p* = 0.014. These data are shown as scatterplots in Supplementary Fig. S3.

The Spearman correlation between mean SW1 trials effects (Fig. 7A) and mean SCR trials effects (Fig. 2A) approached significance, *r*(10) = 0.48, one-tailed *p* = 0.059. Analyses of the individual condition data in Figs. 7B and 2B indicated that this correlation reached statistical significance only in the significant condition at the short ISI, *r*(10) = 0.53, one-tailed *p* = 0.038; see Supplementary Fig. S4.

For the SW2 positivity, the correlation with mean SCR trials effects was not statistically significant, *r*(10) = 0.36, one-tailed *p* = 0.128. Assessment of the individual condition data indicated that this correlation in the significant condition was positive at the short ISI, *r*(10) = 0.55, one-tailed *p* = 0.033, but was non-significant (and negative) at the long ISI, *r*(10) = − 0.49, two-tailed *p* = 0.11. See scatterplots in Supplementary Fig. S5.

## Discussion

The novelty of this study was the joint examination of electrodermal and ERP responses to both indifferent and significant stimuli in auditory dishabituation paradigms presented to two groups at different ISIs. Table 1 presents an overview of the impact of significance and ISI on SCR and all identified components, together with the OR-relevant trials effects in the P300 components.

**Table 1:** Main effects of the independent variables.

Effect	SCR	Na	P1	N1a	N1b	N1c	P2	P3a	P3b	nP3	SW1	SW2
Significance	↑	–	–	~ ↑	†	↓	↑	–	↑	~ ↓	†	–
ISI	↑	–	–	–		~	~ †	–	↑	↑	↓	↓
Decrement	✓							–	✓	✓	–	–
Recovery	✓							–	–	✓	–	–
Dishabituation	✓							–	–	–	–	–

↑Increase; –no effect; ↓Decrease; †interaction; ~ approached significance; ✓observed; Empty cells not tested.

The main aim was to clarify trials effects in the impact of stimulus significance and ISI for the different measures in relation to response *decrement* with stimulus repetition, response *recovery* to a change stimulus, and *dishabituation* (response increase) to the repetition of the standard stimulus following the change stimulus. This basic pattern in response to indifferent stimuli was compared with that to significant stimuli, in a counterbalanced task requiring participants to silently count the stimuli for later reporting to the experimenter.

### SCRs

Averaged over trials, electrodermal responses were larger to significant than to indifferent stimuli, and to stimuli presented at long than short ISIs, with no interaction. The enhancing effect of stimulus significance in the OR has been noted since the early work by Sokolov^1^ and commonly reported in the literature^23,38,39^. The main effect of the between-group difference in ISI is relatively novel, but it is compatible with previous findings by Recio and colleagues^17^. Importantly, there was no interaction between stimulus significance and ISI, suggesting the independence of the two experimental variables.

In the current study, across significance and ISI, the SCR showed response decrement, recovery at the stimulus change, followed by dishabituation, confirming the classic response profile defining a decremental process as habituation^4,5^. The habituation profile in the electrodermal response has long been reported^1,2^. Here, SCR showed the predicted main effects of ISI, significance, and repetition/change, which define the OR.

Importantly, greater decrement over trials was apparent for SCRs to significant stimuli, and this was attributed to the larger initial responses at the first trial. This faster decrement is contrary to the Sokolovian concept that responses to significant stimuli habituate more slowly than those to indifferent stimuli^1,23^. The greater initial OR to significant stimuli across ISI groups is most likely attributable to a heightened arousal state to facilitate the direction of attention towards the counting task^40^. This heightened arousal likely played a role in the ISI effects as we observed greater decrement for responses in the short compared to the long ISI group, particularly in the significant condition. These aspects of the decremental profile need exploration in future studies. Further, recovery at the change stimulus showed the same pattern as the decrement rate: larger for significant than indifferent stimuli, and for the short ISI group, particularly in the significant condition, suggesting that decrement over trials and recovery at a change stimulus are driven by similar or comparable mechanisms. However, dishabituation to the following stimulus did *not* differ significantly with the experimental variables, showing only some enhancement at the short ISI. The small effect sizes suggest that the experimental variables have little functional effect on dishabituation of SCR amplitudes. This would naturally imply a difference in the mechanism between the phenomena of *decrement/recovery* and *dishabituation* that goes beyond novelty processing^13,14,22^. We will return to this later.

### ERPs

The four sets of ERPs, across all trials for both conditions and ISIs had similar components in our separate PCAs: Na, P1, N1a, N1b, N1c, P2, P3a, P3b, nP3, SW1, and SW2. Components from P1 to SW1 closely matched the latency and topography of those reported in habituation paradigms by MacDonald and Barry^38^, and Study 1 of Barry and colleagues^39^. A similar Na at 29 ms was reported in two dishabituation studies with very long ISIs (50–70 s)^36,37^. The SW2 seen here had not been extracted previously using shorter epochs as input to tPCA. The latencies of the components found here were highly correlated across the four datasets. Latencies of components from significant stimuli were significantly longer than for indifferent stimuli; this was most apparent in N1b, N1c, P2, P3a, P3b, and nP3. This might reflect the engagement of numerical cognitive processes due to the counting of stimuli (either via direct retrieval or an automated counting process)^41^. Note also the somewhat condition-specific topographies for some components (e.g., N1c, P3a, P3b, nP3, SW1, SW2) evident in Fig. 4, supporting this notion of differential neurocognitive processing that has previously been linked with the left inferior parietal cortex^42^. These possibilities are beyond the scope of the present study, but deserve future exploration. There was no significant latency difference between matched components in the different ISI groups.

Across trials, amplitudes of Na and P1 were independent of the experimental variables. In significant compared to indifferent conditions, P2 and P3b were larger, while N1c and nP3 were smaller. At very long ISIs (50–70 s) MacDonald and Barry^38^ had reported enhancements to significant stimuli in N1a, P2, and SW1; the differences here are probably due to the much shorter ISIs. N1a was somewhat enhanced at long ISI. Main effects of longer ISI were apparent in significant increases in P3b and nP3, replicating the P300-ISI effect^25–31^, but reductions in SW1 and SW2 were observed.

There were some interactions for ERP component amplitudes between significance and ISI. Although N1b showed no main effects, its amplitude was significantly larger to significant stimuli at the long ISI. The N1c reduction to significant stimuli was somewhat larger for long ISIs, but the nP3 reduction to significant stimuli was significantly larger at short ISIs. The SW1 enhancement at short ISIs was larger for significant stimuli. While such interactive effects are important in adding to our general knowledge about the determinants of different ERP components, they are not our major focus here and will not be discussed further.

### P300 component trials effects

Trials effects in the components forming the P300 complex^9^ were examined and compared to those in the SCR OR model, building on our earlier work^34^. Across significance and ISI, P3a did not decrease linearly over trials, but significant stimuli produced a faster initial decrement (quadratic trend) and the linear decrement was larger for significant stimuli at short ISIs. In P3b, the linear decrement was significant across conditions and ISI, and was somewhat larger for significant than indifferent stimuli. With nP3, a linear decrement was significant across all data sets, and larger for indifferent than significant conditions, compatible with Sokolovian predictions for the OR^1,23^, but contrary to the SCR finding. For SW1 positivity, the linear decrement over trials was somewhat larger for significant than indifferent stimuli. The linear decrement for the SW2 positivity was greater at the short ISI, particularly in the significant condition. Recovery at the change stimulus was not apparent in P3a; indeed, a significant reduction was apparent instead. P3b showed recovery only with significant stimuli, indicating its sensitivity to stimulus relevance^43^. With nP3 and SW1, similar enhanced responding was apparent for all conditions and ISIs; SW2 showed no recovery. No LPC component showed evidence of dishabituation.

In summary, across significance and ISI, P3a, SW1 and SW2 did not decrement significantly, while P3b and nP3 showed significant linear decrement. An increase at the change trial was apparent in nP3 and SW1; the latter effect strictly should not be termed “recovery” as it did not follow decrement over repeated standards. That is, only nP3 showed a true sensitivity to stimulus novelty with decrement and recovery across all experimental conditions, matching the response profile of SCR. This replicates the prior findings of Barry and colleagues^9^. Variations in this mean profile in response to significance (e.g., enhanced decrement in P3b [like SCR], and reduced decrement in nP3) suggests their differential sensitivity to other mechanisms.

There was no evidence of dishabituation in any P300 subcomponent. This finding confirms the ERP results of the three parametric dishabituation studies summarised in MacDonald and Barry^44^. Those data led to the suggestion that dishabituation should “be abandoned as an aspect of habituation” (P. 125), and this is supported here for our ERP components. Our findings in SCR suggested above that a different mechanism is involved in decrement/recovery versus dishabituation, and together with the P300 component results, argue for a wider re-examination of the validity of this habituation criterion in humans (*cf*. Rankin and collegues^5^, and Thompson and Spencer^4^).

### SCR/P300 component comparisons

The correlation analyses indicated that the mean LPC component pattern over all trials compared with that of the SCR did not approach significance for P3a, approached significance for P3b, was significant and strong for nP3, approached significance for SW1, but was not significant for SW2. In addition, the exploratory analyses showed trials effects linked to SCR for P3a in the significant condition at the long ISI, P3b in the significant condition at both ISIs, and with SW1 and SW2 in the significant condition at the short ISI. For nP3, these links were for the indifferent condition at both ISIs and in the significant condition at the short ISI. Overall, these results indicate that the various P300 components have complex relations with SCR as regards their response over trials to stimulus repetition and change, stimulus significance, and ISI. These novel findings need exploration in future studies varying stimulus presentation parameters such as intensity and ISI jointly.

The SCR OR profile and its relationships to the stimulus parameters tested here provided a model for comparison with the P300, long touted as the likely ERP correlate of the OR. In numbers of studies^8,10,34–38,44^ we have repeatedly found that temporal PCA extracted P3a, P3b, nP3, and SW1 components from the P300, and we focussed here on these as potential OR measures. Using a longer ERP analysis epoch here, we also examined a later SW2 than previously discussed. Across trials, increased stimulus significance did not affect P3a, but increased P3b, reduced nP3, and had no main effect on SW1 or SW2; longer ISI had no effect on P3a, but increased P3b and nP3, and decreased SW1 and SW2. Across significance and ISI, P3a, SW1 and SW2 did not decrement, while P3b and nP3 did; recovery to a change following decrement was shown by nP3 only. No components showed dishabituation here. These results confirm the suggestion of MacDonald and Barry^44^ that none of the P300 components mirror SCR, but leave open the possibility that their totality in the P300 may serve as the central analogue of the SCR OR measure, as previously suggested^13,18^.

## Methods

### Participants

Forty undergraduate students participated in this study in return for course credit (mean age = 19.3, *SD* = 1.6 years; 36 right-handed, 24 males). All provided informed consent prior to participating and were free to withdraw at any time without penalty. Participants self-reported no use of psychotropic medication, no neurological or psychiatric illnesses, refrained from psychoactive substances for at least 12 h, and from tea, coffee, alcohol, and cigarettes for at least 2 h prior to testing. All participants had normal or corrected-to-normal vision and self-reported normal hearing.

### Ethics statement

All experimental procedures were carried out in accordance with relevant guidelines and regulations in a protocol approved by the joint South Eastern Sydney/Illawarra Area Health Service and University of Wollongong Health and Medical Human Research Ethics Committee. Participants were informed about the experiment and told that they were free to withdraw at any time without penalty; all provided written informed consent.

### Procedure

Participants completed a demographic and screening questionnaire before they were fitted with EEG and skin conductance recording apparatus. They were seated in a darkened air-conditioned room 60–80 cm in front of a 48.3 cm (19 inch) Dell LCD monitor. Prior to the experiment, participants completed an electrooculogram (EOG)/EEG calibration task^45^. Participants were then instructed to fixate on a 10 × 10 mm grey cross centred on a black background during the task.

Stimuli consisted of 1000 and 1500 Hz tones (counterbalanced between subjects), each of 50 ms duration (15 ms rise/fall time) at 60 dB SPL, delivered binaurally through Sony MDR V700 circumaural stereo headphones. The stimulus sequence was 10 standards of one frequency, a deviant tone of a different frequency, followed by 2–4 standards of the original frequency. Half the participants (*N* = 20) received a short-ISI version of the task (5–7 s SOA), and half (*N* = 20) received a long-ISI version (13–15 s SOA). All participants completed two counterbalanced conditions presented approximately 3 min apart: indifferent, in which there was no task in relation to the auditory stimuli; and significant, where participants were directed to silently count the tones and report the number to the researcher at the end of the condition.

### Material and apparatus

Electrodermal data were recorded from the distal volar surface of digits II and III of the non-dominant hand using sintered silver/silver-chloride (Ag/AgCl) electrodes filled with isotonic electrode paste of 0.05 M NaCl in an inert ointment base. Skin conductance was sampled using a constant voltage device (UFI Bioderm model 2701) at 0.5 V.

Continuous EEG data and DC-coupled skin conductance output were recorded DC–30 Hz at 1000 Hz with a Neuroscan Synamps 2 digital signal-processing system and Neuroscan 4.3.1 Acquire software using the default gain setting, and stored on a Dell Optiplex 755 computer. EEG data were acquired from 19 scalp sites (Fp1, Fp2, F7, F3, Fz, F4, F8, T3, C3, Cz, C4, T4, T5, P3, Pz, P4, T6, O1, O2) with an electrode cap using tin electrodes placed in accordance with the International 10/20 System^46^, plus A1 and A2. Over the duration of this two-group study, some aspects of the protocol were modified to keep up with changing recommendations. In particular, physically-linked ears (with carefully-balanced impedances) were used as a reference for the short-ISI group, and A1 was used for the long-ISI group (later re-referenced to digitally-linked A1/A2); it is not believed that this difference would impact the present findings. The cap was grounded by an electrode located midway between Fp1, Fp2 and Fz. Display and stimulus markers were controlled by a linked stimulus computer using Neurobehavioral Systems Inc. Presentation V 13.0 Build 01.23.09 software.

EOG was recorded using tin cup electrodes placed 2 cm above and below the left eye for vertical movements, and on the outer canthus of each eye for horizontal movements. Impedance was less than 5 kΩ for cap, EOG, and reference electrodes.

### Data extraction

#### Skin conductance response

Raw data were band-pass filtered (0.1–3.0 Hz, zero-phase shift, 24 dB/Octave) and epoched offline 1 s pre- to 5 s post-stimulus using Neuroscan 4.3.1 Edit Software. For each trial, pre-response levels (1 s pre- to 1 s post-stimulus onset) were linearly extrapolated to compensate for falling baselines^47^, using the linear detrend function in Neuroscan. Each response with onset latency 1–3 s post-stimulus^48^ was quantified for each subject and each trial, as the difference between the extrapolated baseline and the maximum value of the subsequent peak^13,14,22,47^. SCRs were square-root transformed to reduce skew^7,23^. Trials that contained non-stimulus related responses were removed and replaced by an average of the available trials immediately before and/or after the aberrant trial.

#### ERPs

EEG data were EOG corrected using the RAAA EOG Correction Program^45^. Where necessary, data were re-referenced to digitally linked ears and extracted offline using the Neuroscan Edit software, low pass filtered (0.1–30 Hz, zero-phase shift, 24 dB/Octave), epoched from 100 ms pre- to 550 ms post-stimulus; single trials were baselined to the pre-stimulus period. Data were manually inspected for any additional artefact, but there were no contaminated trials that required exclusion from analysis.

#### Principal components analyses

The single trial data from − 100 to 550 ms for each of the conditions (indifferent vs. significant) and ISIs (long vs. short) from 19 scalp locations were down-sampled to 500 Hz and submitted to four separate temporal PCAs to reduce misallocation of variance^49^. PCAs were derived in MATLAB (The Mathworks, R2012b Version 8.0.0.783) using Kayser and Tenke's^50^ erpPCA and Varimax4M functions, with a heuristic modification as reported in Barry and colleagues^49^ based on Dien’s^51^ approach of *not* removing the grand mean ERP waveform from each case before calculating the final component waveforms. Factors were quantified separately for each group (4,560 observations: 20 participants × 12 trials × 19 sites). The PCAs used the unstandardised covariance matrix with Kaiser normalisation, and all 325 unrestricted factors underwent Varimax rotation, following Kayser and Tenke^50^. We considered the components ranked in variance order, expecting components in the existing ERP literature. We initially examined all components carrying more than 2.0% variance, to avoid omitting any substantive components, and this threshold was lowered to seek corresponding components if a component was identified in one group or condition only. Component amplitudes were formed by multiplying the factor loading × factor score × standard deviations of the variables.

### Statistical analyses

For each variable, *z* scores (over condition, ISI, and trial) were used to detect univariate outliers (|*z*|≥ 2.5, *p* < 0.01) following Ho's^52^ recommendations for small sample sizes (*N* ≤ 80). Data transformations increased the number of outliers and given the limited sample size (*N* = 20 per ISI group), nature of the data (12 trials within-subjects/conditions), and low percentage of outliers (1.38% of all datapoints), outlier scores were *rescaled* (rather than *omitted*) to avoid participant loss and maintain statistical power^53^. Each identified outlier was individually proportionately rescaled to meet the criterion for retention (|*z*|< 2.5). Across the 11,520 data points (20 participants × 2 groups × 2 conditions × 12 trials × 12 variables), 159 adjustments were required; within each variable, no more than 1.8% (17 of 960) individual datapoints were rescaled, and the mean scaling factors ranged from 0.68 to 0.87 (*M* = 0.79, *SD* = 0.05).

Mixed univariate MANOVAs assessed the between-subjects effect of ISI (long vs. short), and the within-subjects effect of significance (indifferent vs. significant) across all 12 trials, separately for SCRs and ERP components. Separate mixed univariate MANOVAs were used to examine within-subjects response decrement (trial factor) for SCRs and selected ERP components (linear and quadratic trends over trials 1–10), with factors of ISI and condition. Separate mixed univariate MANOVAs were carried out to assess response recovery (trial 11 vs. 10) and dishabituation (trial 12 vs. 10), again with factors of ISI and significance. All *F* tests had *df* = (1, 38).

Correlations were used to compare the grand mean SCR pattern over trials with corresponding patterns in the P300 components. The SCR trials data violated the assumption of normality, so Spearman’s Rank Order correlations were used. Positive correlations were expected to indicate potential OR measures and were tested with one-way significance levels with alpha set to 0.05; all other correlations are reported as two-tailed tests.

It should also be noted that, as this paper details results for a number of dependent measures, the frequency of Type I errors increases. However, Howell^54^ argues that this increase in frequency of Type I errors cannot be controlled by adjusting α-levels because the probability of Type I error remains unchanged.

### Supplementary Information

Supplementary Information.

## Data Availability

All data used in this study are available from the corresponding author on reasonable request.

## References

[1] Sokolov EN (1963). Perception and the Conditioned Reflex.

[2] Sokolov EN (1963). Higher nervous functions: The orienting reflex. Annu. Rev. Physiol..

[3] Sokolov, E. N. The orienting reflex, its structure and mechanisms. In *Orienting Reflex and Exploratory Behavior* (Eds. Voronin, L. G., Leontiev, A. N., Luria, A. R., Sokolov, E. N. & Vinogradova. O. S.). (1965).

[4] Thompson RF, Spencer WA (1966). Habituation: A model phenomenon for the study of neuronal substrates of behavior. Psychol. Rev..

[5] Rankin CH (2009). Habituation revisited: An updated and revised description of the behavioral characteristics of habituation. Neurobiol. Learn. Mem..

[6] Macefield VG, Wallin BG (1996). The discharge behaviour of single sympathetic neurones supplying human sweat glands. J. Auton. Nerv. Syst..

[7] Barry RJ, Sokolov EN (1993). Habituation of phasic and tonic components of the orienting reflex. Int. J. Psychophysiol..

[8] Barry RJ, MacDonald B, Rushby JA (2011). Single-trial event-related potentials and the orienting reflex to monaural tones. Int. J. Psychophysiol..

[9] Barry RJ (2020). Components in the P300: Don’t forget the Novelty P3!. Psychophysiology.

[10] Rushby JA, Barry RJ (2009). Single-trial event-related potentials to significant stimuli. Int. J. Psychophysiol..

[11] Alexander DM (2005). Separating individual skin conductance responses in a short interstimulus-interval paradigm. J. Neurosci. Meth..

[12] Breska A, Maoz K, Ben-Shakhar G (2011). Interstimulus intervals for skin conductance response measurement. Psychophysiology.

[13] Steiner GZ, Barry RJ (2011). Pupillary responses and event-related potentials as indices of the orienting reflex. Psychophysiology.

[14] Steiner GZ, Barry RJ (2014). The mechanism of dishabituation. Front. Integr. Neurosci..

[15] Barry RJ, Rushby JA (2006). An orienting reflex perspective on anteriorisation of the P3 of the event-related potential. Exp Brain Res..

[16] Lim CL (1999). Dynamics of SCR, EEG, and ERP activity in an oddball paradigm with short interstimulus intervals. Psychophysiology.

[17] Recio G, Schacht A, Sommer W (2009). Effects of inter-stimulus interval on skin conductance responses and event-related potentials in a Go/NoGo task. Biol. Psychol..

[18] Rushby JA, Barry RJ, Doherty RJ (2005). Separation of the components of the late positive complex in an ERP dishabituation paradigm. Clin. Neurophysiol..

[19] Donchin E (1984). Cognition and event-related potentials II. The orienting reflex and P300. Ann. NY Acad. Sci..

[20] Gatchel RJ, Lang PJ (1974). Effects of interstimulus interval length and variability on habituation of autonomic components of the orienting response. J. Exp. Psychol..

[21] Berti S, Vossel G, Gamer M (2017). The orienting response in healthy aging: Novelty P3 indicates no general decline but reduced efficacy for fast stimulation rates. Front. Psychol..

[22] Steiner GZ, Barry RJ (2011). Exploring the mechanism of dishabituation. Neurobiol. Learn. Mem..

[23] Barry RJ (2004). Stimulus significance effects in habituation of the phasic and tonic orienting reflex. Integr. Phys. Beh. Sci..

[24] Barry RJ (2009). Habituation of the orienting reflex and the development of preliminary process theory. Neurobiol. Learn. Mem..

[25] Fitzgerald PG, Picton TW (1981). Temporal and sequential probability in evoked potential studies. Can. J. Psychol..

[26] Gonsalvez CJ, Polich J (2002). P300 amplitude is determined by target-to-target interval. Psychophysiology.

[27] Miltner W, Johnson R, Braun C (1991). Auditory and somatosensory event-related potentials: II. Effects of inter-stimulus interval. J. Psychophysiol..

[28] Nakajima Y, Imamura N (2000). Probability and interstimulus interval effects on the N140 and the P300 components of somatosensory erps. Int. J. Neurosci..

[29] Polich J (1990). P300, probability, and interstimulus interval. Psychophysiology.

[30] Polich J (1990). Probability and inter-stimulus interval effects on the P300 from auditory stimuli. Int. J. Psychophysiol..

[31] Polich J, Brock T, Geisler MW (1991). P300 from auditory and somatosensory stimuli: Probability and inter-stimulus interval. Int. J. Psychophysiol..

[32] Ritter W, Vaughan HG, Costa LD (1968). Orienting and habituation to auditory stimuli: A study of short term changes in average evoked responses. Electroencephalogr. Clin. Neuro..

[33] Nieuwenhuis S, De Geus EJ, Aston-Jones G (2011). The anatomical and functional relationship between the P3 and autonomic components of the orienting response: P3 and orienting response. Psychophysiology.

[34] Barry RJ, MacDonald B, De Blasio FM, Steiner GZ (2013). Linking components of event-related potentials and autonomic measures of the orienting reflex. Int. J. Psychophysiol..

[35] Barry RJ, Steiner GZ, De Blasio FM (2016). Reinstating the novelty P3. Sci. Rep..

[36] MacDonald B, Barry RJ, Bonfield RC (2015). Trials and intensity effects in single-trial ERP components and autonomic responses in a dishabituation paradigm with very long ISIs. Int. J. Psychophysiol..

[37] MacDonald B, Barry RJ (2014). Trial effects in single-trial ERP components and autonomic responses at very long ISIs. Int. J. Psychophysiol..

[38] MacDonald B, Barry RJ (2017). Significance and novelty effects in single-trial ERP components and autonomic responses. Int. J. Psychophysiol..

[39] Barry RJ (2022). Stimulus intensity effects and sequential processing in the passive auditory ERP. Int. J. Psychophysiol..

[40] Coull JT (1998). Neural correlates of attention and arousal: Insights from electrophysiology, functional neuroimaging and psychopharmacology. Prog. Neurobiol..

[41] Thevenot C, Barrouillet P (2020). Are small additions solved by direct retrieval from memory or automated counting procedures? A rejoinder to Chen and Campbell (2018). Psychon. Bull. Rev..

[42] Rivera SM, Reiss AL, Eckert MA, Menon V (2005). Developmental changes in mental arithmetic: Evidence for increased functional specialization in the left inferior parietal cortex. Cereb. Cortex..

[43] Verleger R (2020). Effects of relevance and response frequency on P3b amplitudes: Review of findings and comparison of hypotheses about the process reflected by P3b. Psychophysiol..

[44] MacDonald B, Barry RJ (2020). Integration of three investigations of novelty, intensity, and significance in dishabituation paradigms: A study of the phasic orienting reflex. Int. J. Psychophysiol..

[45] Croft RJ, Barry RJ (2000). EOG correction of blinks with saccade coeffcients: A test and revision of the aligned-artefact average solution. Clin. Neurophysiol..

[46] Jasper HH (1958). Report of the committee on methods of clinical examination in electroencephalography. Electroen. Clin. Neuro..

[47] Barry RJ, Feldmann S, Gordon E, Cocker KI, Rennie C (1993). Elicitation and habituation of the electrodermal orienting response in a short interstimulus interval paradigm. Int. J. Psychophysiol..

[48] Barry RJ (1990). Scoring criteria for response latency and habituation in electrodermal research: A study in the context of the orienting response. Psychophysiol..

[49] Barry RJ, De Blasio FM, Fogarty JS, Karamacoska D (2016). ERP Go/NoGo condition effects are better detected with separate PCAs. Int. J. Psychophysiol..

[50] Kayser J, Tenke CE (2003). Optimizing PCA methodology for ERP component identification and measurement: Theoretical rationale and empirical evaluation. Clin. Neurophysiol..

[51] Dien J (2010). The ERP PCA Toolkit: An open source program for advanced statistical analysis of event-related potential data. J. Neurosci. Methods.

[52] Ho R (2013). Handbook of Univariate and Multivariate Data Analysis with IBM SPSS.

[53] Tabachnick B, Fidell L (2018). Using Multivariate Statistics.

[54] Howell D (1997). Statistical Methods for Psychology.

