# Supplementary material for: Effects of interstimulus interval and significance on electrodermal and central measures of the phasic orienting reflex (OR) in a dishabituation task
Source: Sci Rep. 2023 Aug 19;13:13546. doi: 10.1038/s41598-023-40428-7 (PMC10439882; doi:10.1038/s41598-023-40428-7)
Supplement: Supplementary file 1 — Supplementary Information. [file 41598_2023_40428_MOESM1_ESM.pdf]

Supplementary Material

Effects of interstimulus interval and significance on electrodermal and central measures of the phasic orienting reflex (OR) in a dishabituation task

Robert J. Barry[1]*, Genevieve Z. Steiner-Lim[1,2], Adele E. Cave[1], Frances M. De Blasio[1], Brett MacDonald[1]

[1]Brain & Behaviour Research Institute and School of Psychology, University of Wollongong, Wollongong, Australia

[2]NICM Health Research Institute, Western Sydney University, Penrith, Australia

- Corresponding author at rbarry@uow.edu.au

Exploratory correlations

Below are the scatterplots for the exploratory analyses between the P300 component amplitudes and the SCR amplitudes over the 12 stimulus presentations in each combination of ISI (Short/Long) and condition (Significant/Indifferent, abbreviated Sig/Ind).

Each scatterplot indicates component amplitude versus SCR amplitude. Linear trend lines are indicated. Because SCR was not normally distributed over trials, the indicated correlations (*r*) are Spearman correlations, as reported in the manuscript.

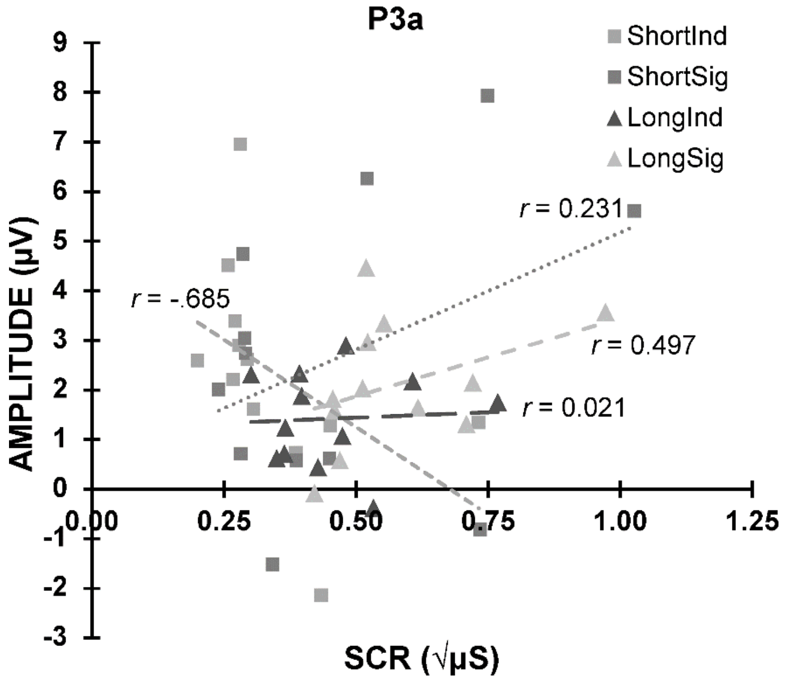

Supplementary Figure S1. Mean P3a component amplitudes plotted against SCR amplitudes at each trial number for the four ISI/Condition combinations.

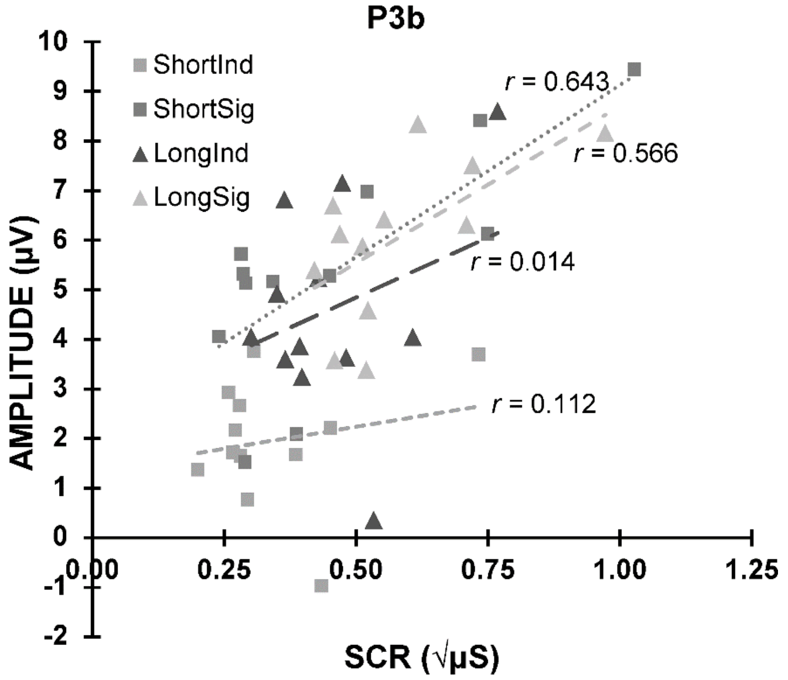

Supplementary Figure S2. Mean P3b component amplitudes plotted against SCR amplitudes at each trial number for the four ISI/Condition combinations.

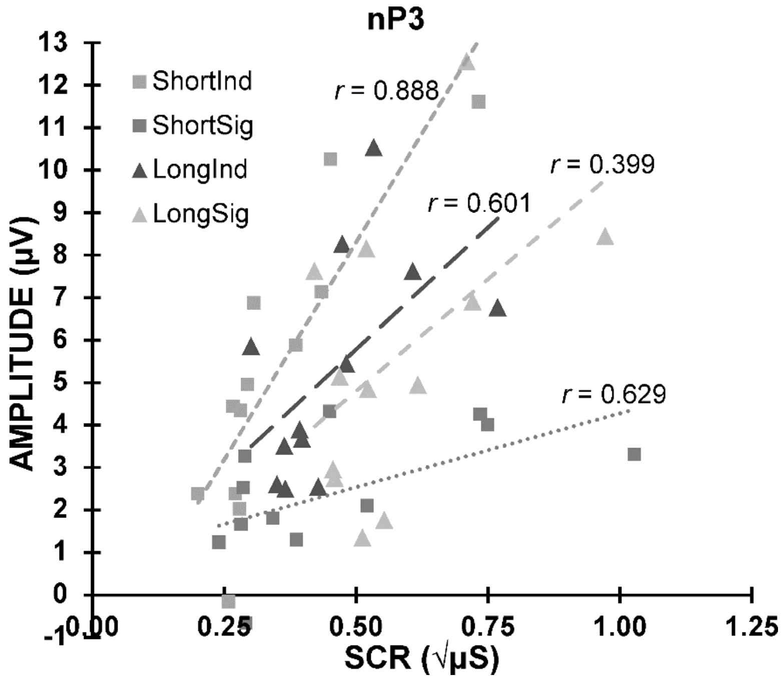

Supplementary Figure S3. Mean nP3 component amplitudes plotted against SCR amplitudes at each trial number for the four ISI/Condition combinations.

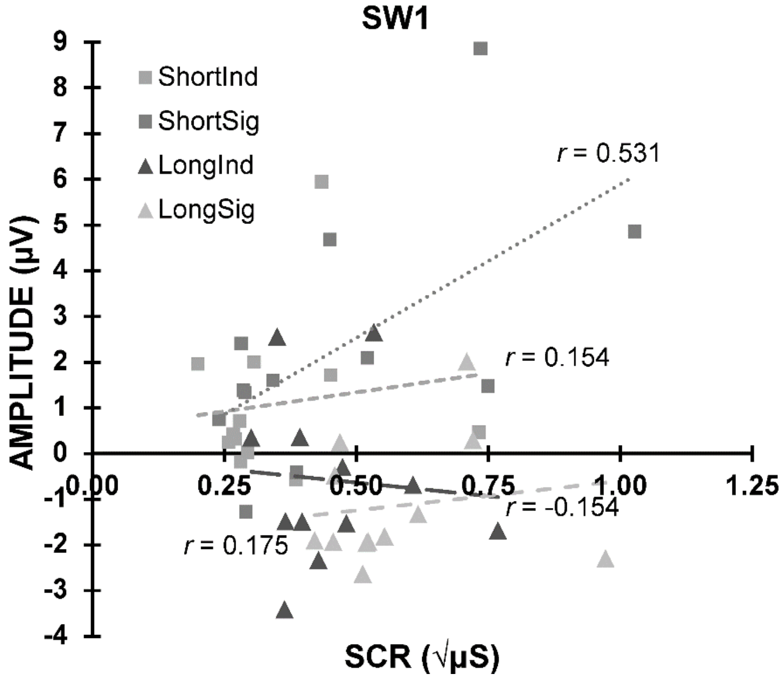

Supplementary Figure S4. Mean SW1 component amplitudes plotted against SCR amplitudes at each trial number for the four ISI/Condition combinations.

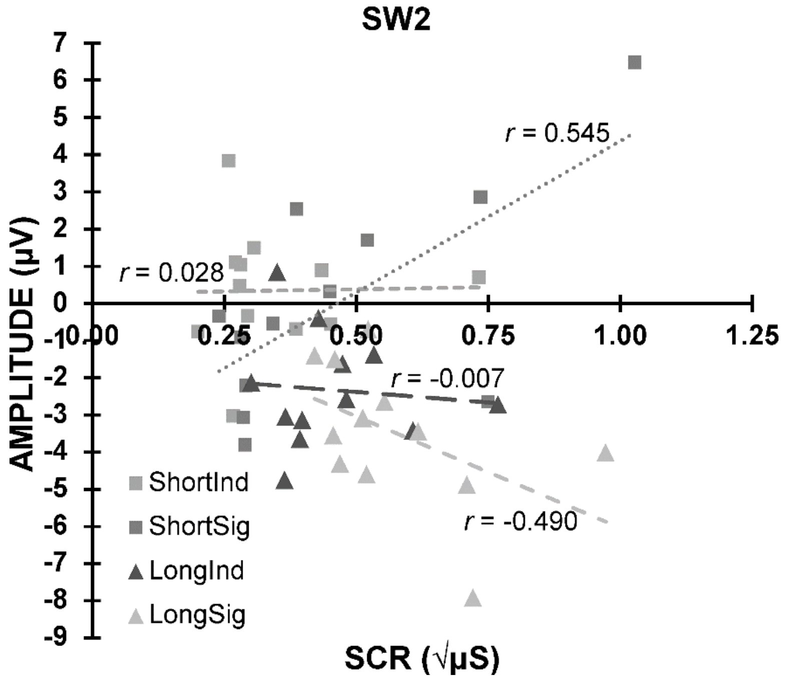

Supplementary Figure S5. Mean SW2 component amplitudes plotted against SCR amplitudes at each trial number for the four ISI/Condition combinations.
